# Single molecule visualization of tropomyosin isoform organization in the mammalian actin cytoskeleton

**DOI:** 10.1002/cm.21883

**Published:** 2024-06-14

**Authors:** Maria L. Cagigas, Nicholas Ariotti, Jeff Hook, James Rae, Robert G. Parton, Nicole S. Bryce, Peter W. Gunning, Edna C. Hardeman

**Affiliations:** ^1^ School of Biomedical Sciences UNSW Sydney Sydney Australia; ^2^ Electron Microscope Unit, UNSW Sydney Australia; ^3^ Institute for Molecular Bioscience The University of Queensland Brisbane Australia; ^4^ Centre for Microscopy and Microanalysis The University of Queensland Brisbane Australia

**Keywords:** actin, cytoskeleton, electron microscopy, isoforms, tropomyosin

## Abstract

The actin cytoskeleton is composed of both branched and unbranched actin filaments. In mammals, the unbranched actin filaments are primarily copolymers of actin and tropomyosin. Biochemical and imaging studies indicate that different tropomyosin isoforms are segregated to different actin filament populations in cells and tissues, providing isoform‐specific functionality to the actin filament. Intrinsic to this model is the prediction that single‐molecule imaging of tropomyosin isoforms would confirm homopolymer formation along the length of single actin filaments, a knowledge gap that remains unaddressed in the cellular environment. We combined chemical labeling of genetically engineered tropomyosin isoforms with electron tomography to locate individual tropomyosin molecules in fibroblasts. We find that the organization of two non‐muscle tropomyosins, Tpm3.1 with Tpm4.2, can be distinguished from each other using light and electron microscopy. Visualization of single tropomyosin molecules associated with actin filaments supports the hypothesis that tropomyosins form continuous homopolymers, instead of heteropolymers, in the presence of all physiologically native actin‐binding proteins. This is true for both isoforms tested. Furthermore, the data suggest that the tropomyosin molecules on one side of an actin filament may not be in register with those on the opposite side, indicating that each tropomyosin polymer may assembly independently.

## 1 INTRODUCTION

The actin cytoskeleton plays multiple vital roles in the cells of most if not all organisms (Pollard & Cooper, 2009). This diversity of function is achieved differently in bacteria, plants, yeast, and animals (Gunning, Ghoshdastider, et al., 2015). Bacteria and plant cells make multiple non‐interchangeable actins to form functionally distinct filaments (Meagher et al., 1999; Popp & Robinson, 2011). In contrast, yeast and animal cells make a relatively small number of different actins, and they achieve functional diversity of most individual linear unbranched filaments by associating the actins with a wide array of tropomyosins (Gunning, Hardeman, et al., 2015).

To form the actin cytoskeleton, actin forms two structurally different types of filaments, namely branched and unbranched filaments (Boiero Sanders et al., 2020). Whereas branched filaments are generally not associated with tropomyosins, most unbranched filaments exist as co‐polymers of actin and tropomyosin in yeast and animals. In these co‐polymers, the tropomyosin molecules form two continuous chains along the major grooves on both sides of the actin filament, resulting in a functionally unique actin/tropomyosin filament (Liu & Bretscher, 1989; Meiring et al., 2018). Each tropomyosin covers 6 or 7 actins in the filament, depending on the isoform, and this raises the question of whether the tropomyosins on each side of the filament are in or out of register with each other.

Historically, tropomyosin has been best understood by its role in regulating the engagement of myosin II motors with unbranched actin filaments to generate contraction of smooth and striated muscle cells (Szent‐Györgyi, 1975). However, more recently, tropomyosin was described as the functional regulator of unbranched actin filaments in the cytoskeleton of multiple cell types, revealing more sophisticated and complex roles than contraction alone. In mammals, over 40 predicted different tropomyosins can regulate filament function in an isoform‐specific manner based on extensive genetic studies (Gunning, Hardeman, et al., 2015; Hardeman et al., 2020). Genetic manipulation of cytoskeletal tropomyosins from yeast to mammals consistently reveals that tropomyosin isoforms within an organism are largely non‐redundant (Hardeman et al., 2020) and the isoform diversity has been maintained in evolution across species (Gunning, Ghoshdastider, et al., 2015).

The mechanism(s) by which tropomyosin isoforms confer functional discrimination between actin filament populations remains a major challenge. Isoforms have been shown to differ in terms of their location in cells and their interactions with myosin motors and actin binding proteins (Bryce et al., 2003; Gunning et al., 2008; Jansen & Goode, 2019; Johnson et al., 2014; Reindl et al., 2022). Recent cryo‐electron microscopy has revealed that two different mammalian tropomyosins sit differently on the actin filament which could at least partially explain the functional differences between these isoforms (Selvaraj et al., 2023). Intrinsic to this interpretation is the assumption that the different isoforms form homopolymers (chains containing the same tropomyosin isoform) in the cell, instead of heteropolymers (chains containing different tropomyosin isoforms). The segregation of isoforms in cells certainly supports the likelihood that the isoforms form homopolymers, however, direct visualization of individual tropomyosin molecules on an actin filament in a cell is lacking (Gunning et al., 2008).

Building on previously reported data showing spatial segregation of tropomyosin isoforms in vitro (Gateva et al., 2017) we hypothesized that individual actin filaments may form homopolymers with a single tropomyosin isoform, giving the filament isoform‐specific identity. Due to the sub‐diffraction limit size of tropomyosins, dissecting the homo‐ or heterogenous composition of the tropomyosin polymers requires super‐resolution or electron microscopy techniques. This study combines electron tomography with endogenous, enzymatically tagged tropomyosins to locate individual tropomyosin molecules along single actin filaments for the first time in mammalian cells. We tested whether the non‐muscle tropomyosin isoforms Tpm3.1 and Tpm4.2 are individually sufficient to saturate actin filaments within the actin cytoskeleton and compared the structural organization of the actin/tropomyosin copolymers at the micro (fiber) and nano (filament) scale.

## 2 MATERIALS AND METHODS

### 2.1 Cells

The mouse line B6‐*Tpm4*tm3(APEX)Hrd (C57BL/6) expressing Tpm4.2 C‐terminally tagged with APEX2 was generated by MEGA (Mouse Engineering at Garvan/ABR) using CRISPR/Cas9. The donor plasmid encoding a linker (sequence GGAGGCGGAGGCTCGGGAGGCGGAGGCTCG) and the ascorbate peroxidase enzyme APEX2 (Meiring et al., 2019; Rae et al., 2021) was knocked in 1 bp upstream of TPM4 exon 9 via homologous recombination. Knock‐ins were confirmed via sequencing. Primary mouse embryonic fibroblasts (PMEFs) were isolated from B6‐*Tpm4*tm3(APEX)Hrd (Tpm4.2‐APEX2) and B6‐*Tpm3*tm6(APEX)Hrd (Tpm3.1‐APEX2) (Meiring et al., 2019) mouse embryos at day 13.5 according to procedures approved by the UNSW Sydney Animal Care and Ethics Committee (Project 17/100B). Procedures were conducted in accordance with the NSW Animal Research Act (1985) and the National Health and Medical Research Council's Australian Code for the Care and Use of Animals for Scientific Purposes (2013). Embryos were genotyped and fibroblasts were isolated, trypsinized and cultured in DMEM (Sigma) supplemented with 10% Fetal Bovine Serum (Gibco) at 37°C with 10% CO_2_.

### 2.2 Western blots

Western blots were performed as previously described (Cagigas et al., 2022). Briefly, Tpm4.2‐APEX2 cells derived from wild‐type and APEX2 homozygous (APEX2/APEX2) embryos were grown until confluency and harvested with RIPA buffer with protease inhibitor (Merck). Lysates were sonicated, mixed with Laemmli buffer (Biorad) with β‐mercaptoethanol (Sigma), and boiled prior to running on polyacrylamide SDS‐PAGE gels. Gels were transferred to PVDF membranes (Millipore), blocked, and sequentially incubated with primary rabbit antibody anti‐Tpm4.2 δ/9d (1:500), secondary goat anti‐rabbit IgG‐HRP (1:3000, BioRad 1,706,515), primary rabbit anti‐α‐tubulin (1:3000, Abcam ab52866), and secondary goat anti‐rabbit IgG‐HRP (1:5000, Biorad 1,706,515). Membranes were washed, incubated with Luminata Crescendo Western HRP substrate (Merk), and imaged (ImageQuant™ LAS 4000 imaging system, GE Healthcare). Band densitometry was quantified in ImageJ and normalized to α‐tubulin controls.

### 2.3 Brightfield and fluorescence microscopy

For brightfield, homozygous Tpm4.2‐APEX2 PMEFs were grown on cell culture dishes, fixed with PFA (Sigma), washed in PBS and diaminobenzidine (DAB), and treated with 1 mg/mL DAB +0.03% H_2_O_2_ for 20 min. Cells were immediately imaged on an Olympus CKX53 brightfield microscope fitted with an Infinity 5 (Teledyne Lumenera) camera using a 20×/0.40 air objective and white light. For IF, tagged and untagged PMEFs were fixed with PFA, permeabilized with 0.2% Triton X‐100 (Scharlau), blocked, and sequentially incubated with the antibodies primary mouse γ/9d 2G10.2 anti‐Tpm3.1/3.2 (1:1000, Merck MABT1335), secondary goat anti‐mouse AF‐488 (1:1000, Invitrogen A‐11001), primary rabbit Tm4/9d anti‐Tpm4.2 (1:1000, 72), and donkey anti‐rabbit AF‐568 (1:1000, Invitrogen A21206), and labeled with Phalloidin‐Atto 647 (1:1000, Sigma). IF protocol as described in Meiring et al. (2018). Cells were imaged on a Zeiss 800 Airyscan confocal microscope using a 63× apo oil objective with 488, 568, and 647 nm lasers and Zen Blue 3.1 software acquisition. *N* ≥ 10 cells were imaged per treatment and processed in ImageJ. Z‐stacks were obtained by maximum intensity projection (MIP).

### 2.4 Electron tomography

Cells were grown, processed, and imaged as previously described (Cagigas et al., 2022; Meiring et al., 2019; Rae et al., 2021). The DAB reaction product generated by the APEX2‐labeled proteins was converted to gold particles as described previously by Rae et al. (2021). Resin sections were imaged in a JEOL JEM‐1400 transmission EM at 120 kV fitted with a Phurona camera (EMSIS GMBH) to obtain an overview of the Tpm4.2‐APEX2 product on actin bundles. Electron tomograms of the same filaments were acquired in a Talos Arctica microscope (ThermoFisher) at 200 kV operating at RT fitted with a Falcon 3EC camera. Tomograms were reconstructed in IMOD using weighted back‐projection.

### 2.5 Analysis of actin/tropomyosin filaments

Single filaments, defined as uninterrupted filamentous densities of width 6–12 nm (thickness criteria defined by the thickness of an individual actin filament plus additional density added to the fiber by EM processing and post‐staining) and between 100 and 300 nm long were identified in the tomograms (IMOD) and between 4 and 6 slices comprising the whole filament were extracted and Z‐projected for analysis (ImageJ). Line‐scans 10 nm thick were manually drawn along the length of the actin filaments, and the intensity profile of the black APEX2 signal was obtained using the Multiplot command (ImageJ). Normalized signal intensity (0–1) was plotted against filament length (nm) to measure peak‐to‐peak distances in individual filaments (Excel). For confocal images, line‐scans were manually drawn along the length of the actin filament, and the fluorescence intensity for Tpm3.1 and Tpm4.2 were obtained using the Multiplot command (ImageJ).

## 3 RESULTS

### 3.1 Homozygous Tpm4.2‐APEX2 knock‐in mice are viable but have reduced expression levels

Recently, APEX‐tagged protein constructs and nanobodies have made the visualization of intracellular proteins in their cellular environment at high resolution possible using transmission electron microscopy (TEM) (Rae et al., 2021). APEX tags also provide the opportunity to label endogenous proteins by knocking the tag into genes of interest in cells or animals. To take advantage of this approach, we genetically engineered APEX2 into the C‐terminus of the cytoskeletal tropomyosins Tpm4.2 and Tpm3.1 in the mouse genome. A 10 amino acid flexible linker was used to minimize the impact of the APEX2 tag on the head‐to‐tail polymerization of tropomyosin along the length of actin polymers. The Tpm3.1‐APEX2 mouse and derived PMEFs were previously characterized (Meiring et al., 2019). This experimental strategy provides the opportunity to identify the association of single tropomyosin isoforms along the length of an actin filament visualized by electron microscopy. However, it is important to note that tropomyosin associates with actin filaments as a dimer and we cannot discriminate in this approach between a homodimer of the tagged tropomyosin and a heterodimer composed of the tagged tropomyosin dimerized with an untagged other isoform.

**FIGURE 1:**
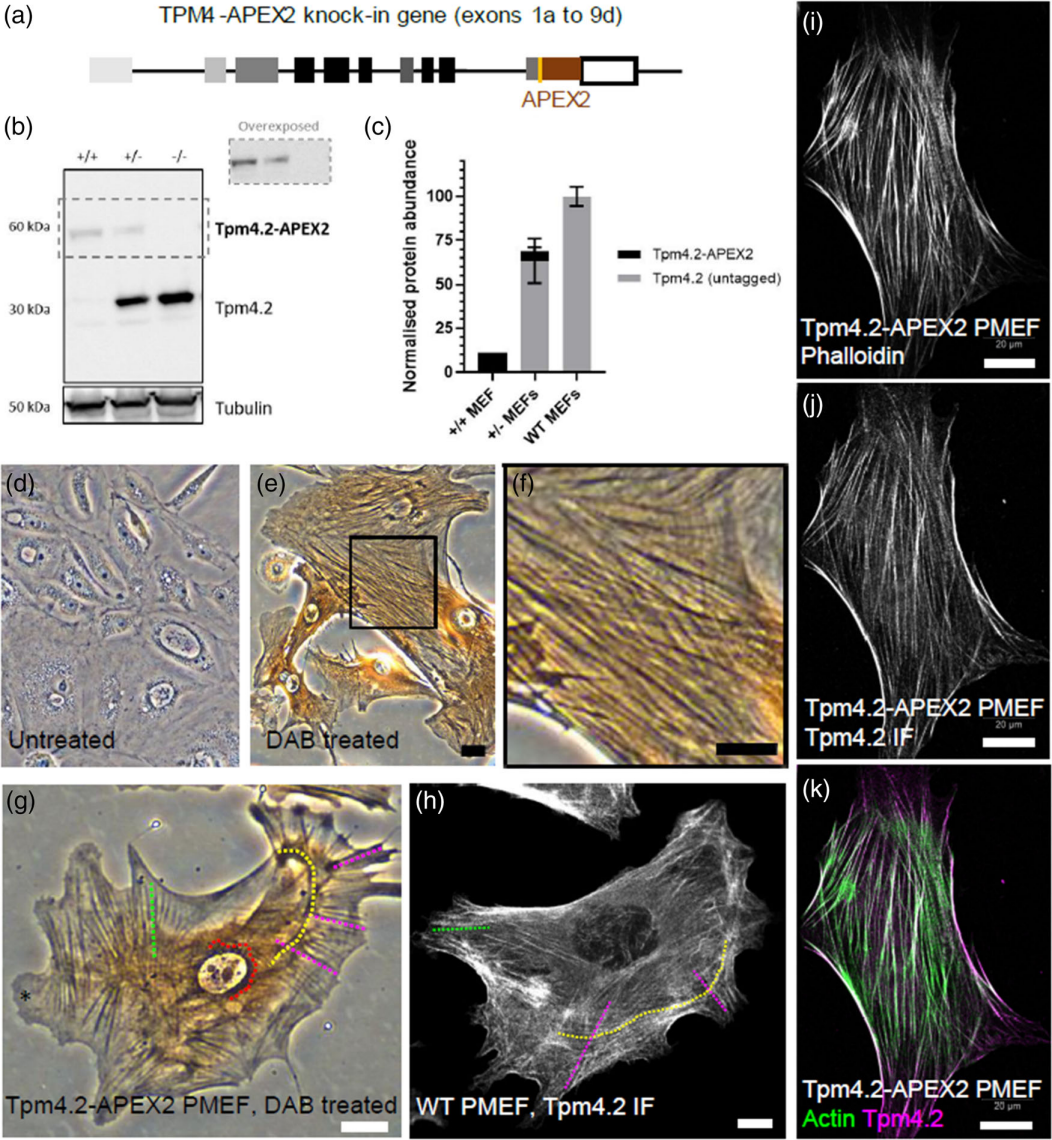
Knock‐in of APEX2 in genetically modified mice allows visualization of tropomyosin 4.2 in stress fibers via APEX2 oxidation of DAB. (a) Schematic representation of the TPM4 gene, modified to express APEX2 tagged to exon 9d in knocked‐in mice. (b) Western blot of PMEF cell lysates (Tpm4.2‐APEX2 homozygous +/+, Tpm4.2‐APEX2 heterozygous +/−, and untagged WT −/−) probed with δ/9d (Tpm4.2) and α‐tubulin (control) antibodies. The overexposed dotted area for Tpm4.2‐APEX2 is shown for clarity. Representative blot from *n* = 9 embryos. (c) From b, protein quantification (mean ± SD). (d, e) Brightfield images of (d) untreated (control) and (e) DAB+H_2_O_2_‐treated Tpm4.2‐APEX2 PMEFs. (f) Zoom‐in area (square in e) showing positive DAB reactions (dark brown) localized to stress fibers. (g) Brightfield image of DAB+H_2_O_2_ treated Tpm4.2‐APEX2 PMEF showing distinct localization of Tpm4.2‐APEX2‐DAB to stress fibers, including transverse arc (yellow dotted line), dorsal stress fibers (magenta dotted line), and ventral stress fibers (green dotted line). Positive DAB reaction is absent from lamellipodia (*) known to exclude Tpm4.2. Perinuclear localization (red dotted line), likely due to unspecific accumulation of DAB in large cytoplasmic volumes, is present. (h) Confocal image of untagged WT PMEF immunolabeled with δ/9d (Tpm4.2) antibody showing native localization of Tpm4.2 to stress fibers, including transverse arc (yellow dotted line), dorsal stress fibers (magenta dotted line), and ventral stress fibers (green dotted line). Localization of Tpm4.2 to stress fibers coincides with tagged (g) and WT (h) PMEFs. Localization to perinuclear actin is absent. (i–k) Confocal images of Tpm4.2‐APEX2 PMEF stained with phalloidin (i), immunolabeled with δ/9d (Tpm4.2) antibody (j), and merge (k) showing co‐localization of Tpm4.2 and actin in stress fibers. Scale bars = 10 μm for panels e–h and 20 μm for panels i–k. DAB, diaminobenzidine; PMEF, primary mouse embryonic fibroblasts.

The structure of the Tpm4.2‐APEX2 knock‐in allele is shown in Figure 1a. Homozygous Tpm4.2‐APEX2 PMEFs (+/+), heterozygous Tpm4.2‐APEX2 PMEFs (+/−), and wild‐type (WT) untagged PMEFs (−/−) were isolated from mice embryos and characterized using western blot (Figure 1b,c) and microscopy (Figure 1d–k). Western blots of Tpm4.2‐APEX2 cell lysates revealed a band at approximately 60 kDa corresponding to the expected size of the tagged Tpm4.2 (Figure 1b). As expected, homozygous cells expressed no untagged Tpm4.2 and the level of the expressed tagged Tpm4.2‐APEX2 was reduced to 10% of the level of endogenous Tpm4.2 in wild‐type cells (Figure 1b,c). This level of reduced expression of the tagged protein is consistent with the previously reported relative expression of Tpm3.1‐APEX2 to endogenous Tpm3.1 (Meiring et al., 2019), and it does not interfere with the incorporation of Tpm4.2 into stress fibers (Figure 1e–k).The similarity of reduced protein accumulation for both the Tpm4.2 and the Tpm3.1/2 APEX2 hybrids indicates that this is not specific to a single isoform nor the chromosome location. Rather, it is consistent with the impact of APEX2 on protein stability although an impact on mRNA stability or translation cannot be ruled out.

### 3.2 Tpm4.2‐APEX2 is incorporated into actin filament bundles in PMEFs

To verify whether Tpm4.2‐APEX2 incorporates into actin filament bundles and retains enzymatic activity, Tpm4.2‐APEX2 PMEFs were treated with DAB and H_2_O_2_ and imaged by brightfield microscopy (Figure 1d–f). The enzyme APEX2 oxidized the DAB generating a dark brown reaction product that abundantly localized to stress fibers (Figure 1e,f). The localization of APEX2‐tagged Tpm4.2 to stress fibers was noticeably similar to the localization of native Tpm4.2 in WT immunolabeled cells (Figure 1g,h) and in previously reported U2OS cells, including an abundance of Tpm4.2 in transverse arcs, dorsal, and ventral stress fibers, and an exclusion from lamellipodia (Tojkander et al., 2011). In contrast, control untreated cells showed no reaction (Figure 1d). Equally, homozygous Tpm4.2‐APEX2 cells immunolabeled with anti‐Tpm4.2 antibody and stained with phalloidin (filamentous actin) showed co‐localization of Tpm4.2 and F‐actin to stress fibers (Figure 1i–k). These results suggest that the APEX2 tag does not impair tropomyosin participation in filament assembly, and, importantly, linkage to tropomyosin and incorporation into filament bundles does not impair APEX2 enzyme activity.

### 3.3 Tpm4.2‐APEX2 is detected in PMEF actin filament bundles using electron tomography

**FIGURE 2:**
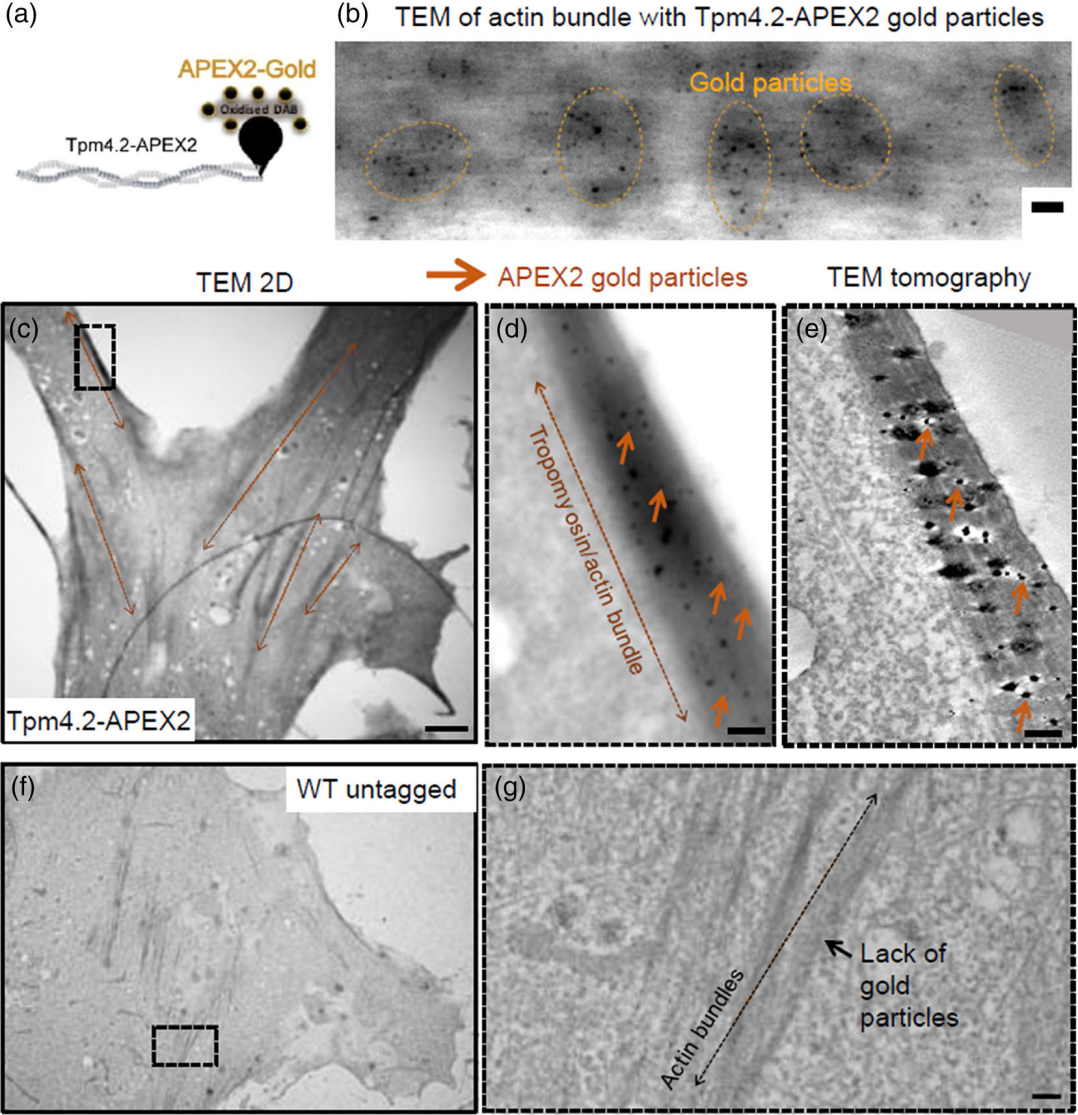
Gold particles nucleated in situ at DAB oxidation sites allow visualization of Tpm4.2 molecules in actin bundles using electron tomography. (a) Schematic representation of a Tpm4.2 molecule genetically tagged to the APEX2 enzyme that oxidizes DAB. Oxidized DAB reduces silver ions that get substituted by gold particles nucleated using tetrachlorogold acid trihydrate. (b) TEM visualization of an actin bundle in a Tpm4.2‐APEX2 PMEF showing abundant gold particles labeling Tpm4.2 molecules that cluster (orange dotted circles) along the bundle. Scale bar = 100 nm. (c–e) TEM image of Tpm4.2‐APEX2 PMEF labeled with chemically generated gold particles at the Tpm4.2 C‐terminal end. Dotted arrow lines show actin bundles with a dark appearance due to the DAB reaction. For comparison, actin bundles in a WT untagged cell (f) are lighter in color and lack gold particles. Scale bar = 2 μm. (d) Zoom‐in of an actin bundle (dotted rectangle from c) with abundant gold particles (brown arrows) imaged at low resolution. Scale bar = 200 nm. (e) Same zoom‐in area as in (d) imaged with high resolution using tomography. Gold particles labeling Tpm4.2 molecules coincide in both images, acquired with different microscopes (JEOL JEM‐1400 at 120 kV and Talos Arctica at 200 kV). Tomography resolves actin filaments within the bundle. Scale bar = 200 nm. (f, g) Untagged cells show lighter actin bundles (f), that lack gold particles (g, zoom‐in from f). Scale bar = 200 nm. DAB, diaminobenzidine; PMEF, primary mouse embryonic fibroblasts; TEM, transmission electron microscopy.

Electron microscopy of Tpm4.2‐APEX2 cells confirmed that this tropomyosin abundantly populates F‐actin bundles (Figure 2). Post DAB treatment, electrodense gold particles were generated via a chemical reaction in situ at the Tpm4.2 C‐termini (Figure 2a). Gold particles labeled Tpm4.2 molecules that abundantly decorated actin bundles preferentially forming clusters (Figure 2b). Low magnification TEM was employed to identify Tpm4.2‐enriched fibers of interest in Tpm4.2‐APEX2 PMEFs (Figure 2c). Gold particles (Figure 2d, brown arrows) served as fiducials to locate and image the actin/tropomyosin filaments at high resolution using electron tomography (Figure 2e). Tomography resolved microtubules, vesicles, cell membranes, and actin filaments (Figure 2e), enabling the study of the distribution of Tpm4.2 in actin filaments. For comparison, untagged WT cells lacking gold particles and dark electrodense staining due to the absence of APEX2 were imaged as a control (Figure 2f,g).

### 3.4 Do Tpm3.1‐APEX2 and Tpm4.2‐APEX2 form continuous copolymers with F‐actin?

**FIGURE 3:**
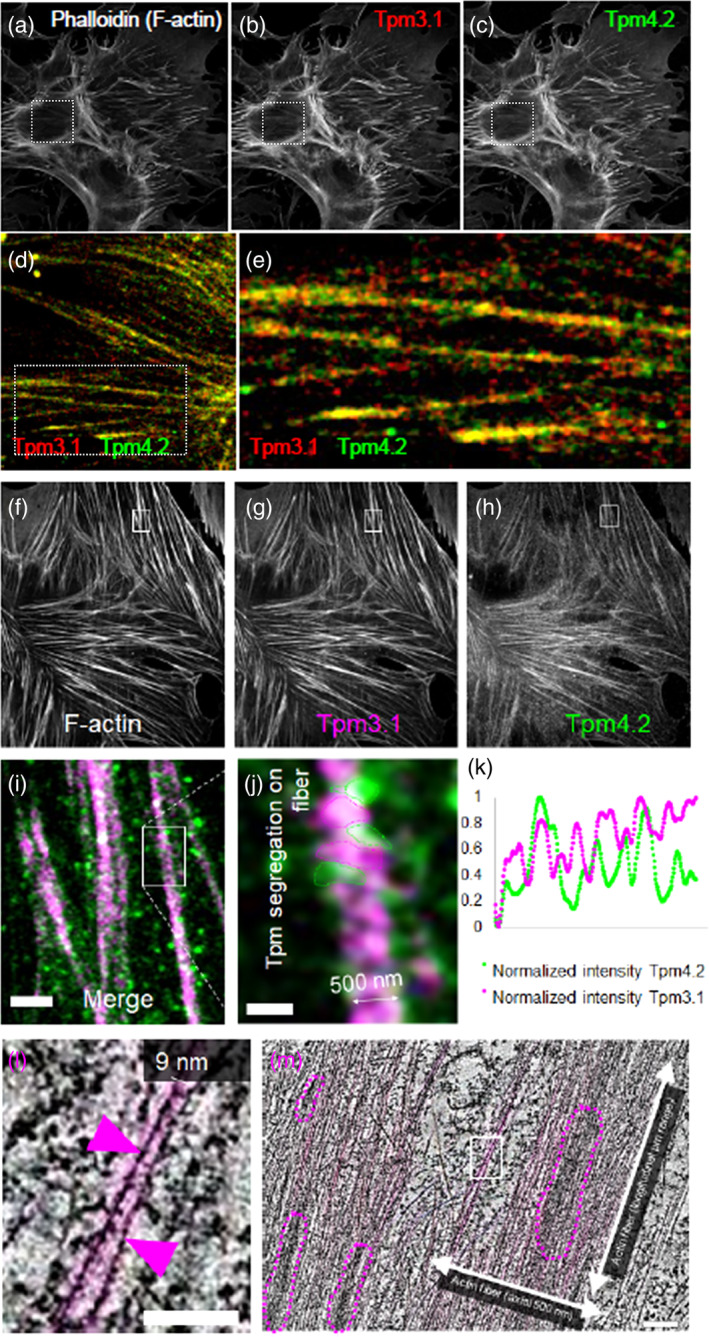
Tropomyosin isoforms 3.1 and 4.2 assemble in clusters along actin bundles. (a) Airyscan confocal images of a PMEF labeled with (a) phalloidin (F‐actin), and antibodies against (b) Tpm3.1 (γ/9d, red) and (c) Tpm4.2 (δ/9d, green). Scale bar = 10 μm. (d) Zoom‐in (dotted square in c) and further zoom‐in (e, dotted rectangle in d) showing individual actin bundles coated by distinct tropomyosin isoforms (Tpm3.1 in red, Tpm4.2 in green). (f–j) Equivalent results in a second PMEF labeled with (f) phalloidin, (g) anti‐Tpm3.1 antibody, and (h) anti‐Tpm4.2 antibody. (i) Zoom‐in (white square in h) of an actin/tropomyosin fiber. Scale bars = 2 μm. (j) Further zoom‐in showing distinct clustering (Tpm3.1 in magenta, Tpm4.2 in green). Scale bar = 500 nm. (k) Line‐scan of normalized fluorescent intensity along an actin bundle for Tpm3.1 (magenta) and Tpm4.2 (green). (L) Z‐projection of 10 TEM tomogram slices showing individually resolved single‐actin filaments (7 nm diameter) decorated by Tpm3.1‐APEX (black signal, magenta arrowheads). Scale bar = 50 nm. (m) TEM tomography of Tpm3.1‐APEX PMEFs showing clustering of Tpm3.1 (black signal), magenta dotted lines) along the actin bundles. Scale bar = 100 nm. PMEF, primary mouse embryonic fibroblasts; TEM, transmission electron microscopy.

Tropomyosin distribution within whole actin bundles shows that Tpm4.2 and Tpm3.1 can co‐populate actin filament bundles at the microscale level (Figure 3a–e,f–k). However, the isoforms are not uniformly distributed and segregate within a filament bundle (Figure 3e,j,k). The same segregation was reported in dorsal stress fibers (Tojkander et al., 2011) and transverse arcs (Meiring et al., 2019) in U2OS cells. Therefore, stress fibers are heterogenous in tropomyosin isoform composition and localization, which potentially allows for isoform dependent tuning of F‐actin‐protein interactions (Meiring et al., 2019; Tojkander et al., 2011). Similarly, actin fibers containing Tpm3.1‐APEX2 imaged using electron tomography (Figure 3m) showed that the oxidized DAB distribution of Tpm3.1 (magenta dotted lines) is not evenly distributed but segregated in clusters along the fiber.

**FIGURE 4:**
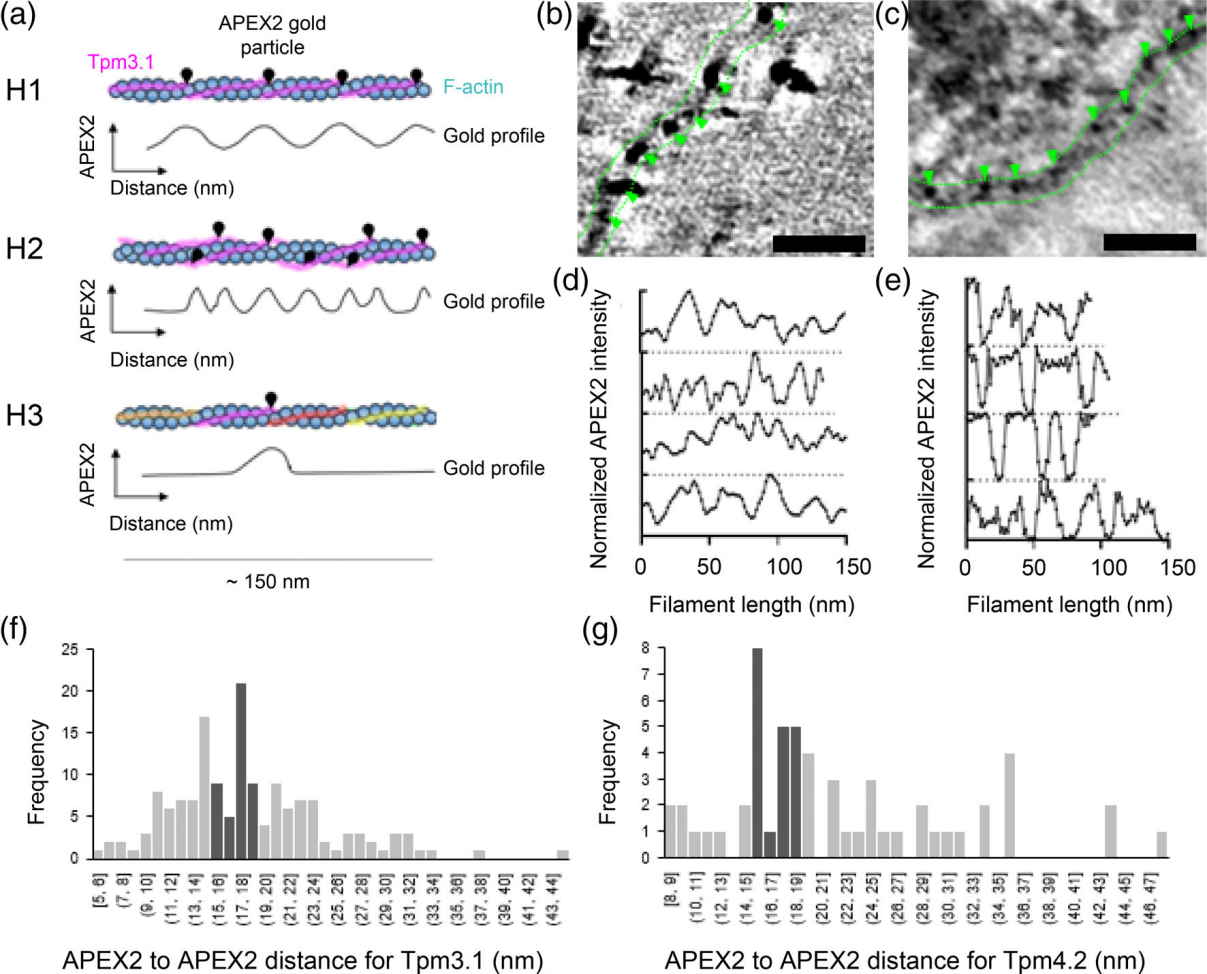
Single tropomyosin isoforms form continuous polymers along individual actin filaments. (a) Schematic H1, H2, and H3 with their predicted APEX2 intensity profiles. Note that this does not assume the signals are due to homodimers since we cannot discriminate homodimers from heterodimers (b) Representative TEM image of individual actin/Tpm3.1 filament (+/+) and (c) actin/Tpm4.2 filament (+/+) labeled with APEX2 gold particles (green arrowheads). Scale bar = 50 nm. (d, e) Representative APEX2‐gold intensity plots along the filament length from *n* = 18 Tpm3.1‐APEX2 filaments (d) and *n* = 14 Tpm4.2‐APEX2 filaments (e). (d, g) Frequency distribution of gold‐particle to gold‐particle distances. Darker columns correspond to 17.5 ± 5% nm distances; TEM, transmission electron microscopy.

Compatible with the finding that tropomyosin isoforms are spatially segregated in a range of cell types and organisms, we hypothesized that the isoforms may form homopolymers with actin at the filament level. We exploited the availability of the APEX2 PMEFs to test whether a single tropomyosin isoform is able and sufficient to saturate individual actin filaments, that are 9 nm in diameter when associated with tropomyosin, in the cell cytoskeleton using electron tomography (Figure 3l, magenta filaments). To analyze the tropomyosin distribution at the filament level it is important to recognize that tropomyosin forms two polymers along both sides of an individual actin filament. We therefore hypothesized that three structural scenarios for the single filament were possible in PMEFs derived from homozygous mice in which all Tpm4.2 or Tpm3.1 molecules are tagged with APEX2: (H1) a single tropomyosin isoform (homopolymer) along one side and possibly both sides with their head‐to‐tail junctions in register, (H2) a single tropomyosin isoform (homopolymer) along both sides with junctions out of register, and (H3) multiple tropomyosin isoforms (heteropolymer) along both sides. The schematic distribution of the APEX2 tag along the filament for each scenario is represented in Figure 4a, where black dots represent the gold particles chemically generated via the APEX2 tag. Since only one tropomyosin isoform (either Tpm4.2 or Tpm3.1) carries APEX2 in each cell type and all the other isoforms are untagged, the corresponding APEX2‐gold intensity profiles for each hypothesis can be differentiated by measuring the distances between adjacent gold particles (H1: periodicity of 36 nm [length of Tpm3.1 and Tpm4.2 is 36 nm]; H2: variable periodicity between 6 and 30 nm; H3: >> 36 nm).

Electron tomography was used to locate single tropomyosin molecules along single actin filaments (Figure 4b–d). The gold particles (green arrowheads) labeling the tropomyosin molecules that form the polymer were arranged periodically along the filaments. To obtain the intensity profiles, line scans were drawn along individual filaments, and the normalized APEX2 signal intensity (corresponding to the gold particles) was plotted against filament length. Figure 4d,e shows the representative graphs for four filaments per condition. The cells consistently displayed periodic tropomyosin labeling (peaks) consistent with each tropomyosin isoform forming a continuous homopolymer along the length of the actin filament.

### 3.5 Are the tropomyosins on either side of the filament out of register?

The periodicity of gold particles provides an insight into the organization of tropomyosin on either side of the filament. Gaps in labeling greater than 36 nm were uncommon (Figure 4f,g). Rather, the distances between the gold tags were variable and mostly smaller than the length of a single tropomyosin consistent with the same tropomyosin isoform present on both sides of the filament (Figure 4f,g). Tpm4.2‐APEX2 cells showed distances smaller than 36 nm 93% of the time (mean 23.4 ± SD 7.5 nm, *n* = 13 filaments); whereas Tpm3.1‐APEX2 did so 99% of the time (mean 18.2 ± SD 6.4 nm, *n* = 19 filaments) (for individual filament measurements, see Supplementary Information). This suggests that the two tropomyosin polymers running along each side of the filament are a best fit with model H2. The distances between APEX deposits rules out H1 and H3 and is only compatible with the same isoform on both sides of the actin with the head‐to‐tail junctions out of register from one side to the other. Direct observation of tropomyosin Tpm1.8 single dimers binding to actin filaments in a cell‐free system reported the same strand independency of one side to the other (Bareja et al., 2020). Overall, we conclude that the best fit with the data is the model where individual isoforms can fully decorate actin filaments in cells forming homopolymers, and that the chain location on either side of the filament relative to each other is flexible.

## 4 DISCUSSION

### 4.1 Tropomyosin isoform segregation as an outcome of forming homopolymers

Studies in multiple organisms have consistently observed the spatial segregation of tropomyosin isoforms (Gunning et al., 2008). This is exemplified by the demonstration in yeast that the acetylated and non‐acetylated isoforms are spatially segregated into actin/tropomyosin homopolymers with contractile and vesicle transport roles respectively (Johnson et al., 2014). Core to these roles are specialized interactions with different myosin motors (Johnson et al., 2014).

Studies in mammals are more challenging because of the large number of tropomyosin isoforms that participate in cytoskeletal assembly. Isoform segregation is measured by comparing the location of different isoforms. The intrinsic weakness with such approaches using either biochemistry or imaging is that aside from the yeast studies, it is not possible to test the location of all isoforms simultaneously due to limitations in labeling. One can therefore never rule out the possibility that an unlabeled isoform co‐segregated with the isoform under study.

An alternative approach to address this issue is to evaluate whether the isoforms form homopolymers. The formation of homopolymers provides both the mechanism that can account for isoform segregation and suggests that even in the most congested filament bundles, the isoforms are segregated to separate filaments. In vitro polymerization of tropomyosin/actin filaments using distinguishable tropomyosin isoforms suggests that the formation of homopolymers is largely intrinsic to the different tropomyosins (Gateva et al., 2017). Furthermore, cryo‐electron microscopy has shown that two different tropomyosin isoforms form polymers on actin filaments with differences in their positioning on the filament which would be likely incompatible with heteropolymer formation (Selvaraj et al., 2023). The power of both these studies is the ability to visualize the organization of tropomyosin on individual actin filaments (Gateva et al., 2017; Selvaraj et al., 2023). Of necessity, both these studies were performed in cell free systems.

The strength of using the APEX2 labeling system in our study is the possibility of dissecting the organization of actin/tropomyosin polymers at the single‐filament level inside a cell, in the presence of the native array of actin binding proteins naturally present in mammalian cells. Three reasons made this challenge previously unattainable, namely (1) actin/tropomyosin filament size is below the sub‐fraction limit of light (9 nm) and filaments are frequently assembled in dense fibers, (2) the high density and suboptimal signal to noise ratio of the cellular environment prevents visualization of label‐free tropomyosin chains using TEM, and (3) the multiple tropomyosin isoforms are indistinguishable from each other without specific tagging, and they often co‐populate the same actin filament bundles. However, our study using high resolution intracellular tomography in association with chemical gold labeling of genetically modified primary cells can partly overcome these limitations, providing the first observation of individual tropomyosin molecules decorating single actin filaments inside cells.

Nevertheless, the approach presents limitations. First, the tropomyosin molecules polymerized along the actin filaments were not seen directly but indirectly via an in situ chemically made electron dense gold particle at the tropomyosin's C‐terminus. Therefore, the approach relies on even access of DAB and H_2_O_2_ to the filaments, active APEX2 enzymes oxidizing the DAB without diffusion, and consistent gold nucleation. To minimize variability, the experimental protocol was thoroughly optimized and repeatedly reproduced in previous studies (Rae et al., 2021). Second, the method was unable to directly distinguish which tropomyosin chain, out of the two chains decorating the opposite grooves of the actin filament, the gold particles belonged to. However, what it did find was that the distance between gold particles was almost always smaller than the size of a tropomyosin molecule (36 nm), suggesting that particles from chains on both sides of the actin filament were being visualized. Lastly, “filament length” was described as the length of the actin filament that was clearly visible and uninterrupted in the electron tomograms so that analysis could be performed (100–300 nm), which did not necessarily equate to the full length of the filament but was sufficient to test the hypotheses.

### 4.2 Tropomyosin organization on each side of actin can be out of register with each other

The measurement of distances between tropomyosin molecules along an actin filament presented in this study is not compatible with the two tropomyosin polymers running along each side of the filament being in register. In addition, some individual APEX2 molecules may not have produced sufficient product to be detected which would result in an overestimation of the average distance between individual tropomyosins. It is a formal possibility that some filaments we imaged were in fact two adjacent filaments that we could not discriminate. However, the consistency of the measurements across different filaments makes this less likely. We therefore propose that the tropomyosin polymers along each side of the filament are not in register for either Tpm3.1 or Tpm4.2.

A similar conclusion has been reached in an in vitro study of the assembly of Tpm1.8 on single actin filaments (Bareja et al., 2020). It was observed that the formation of the Tpm1.8 polymer was not influenced by the existence or register of Tpm1.8 on the other side of the actin filament. Because of the limited resolution of our study, we cannot rule out the possibility that there are small gaps on one side of the filament that further emphasize the lack of register. Nevertheless, the overall consistency with the in vitro data provides a strong argument for the independence of addition of tropomyosin to both sides of the filament.

## 5 CONCLUSIONS

We conclude that single molecule imaging of Tpm3.1 and Tpm4.2 in cells is consistent with the formation of homopolymers of tropomyosin isoforms along the length of an actin filament. Furthermore, our data suggests that the organization of tropomyosin along one side of the actin filament does not influence the organization along the opposite side.

## AUTHOR CONTRIBUTIONS

*Conceptualization and writing*: P.W.G, M.L.C. *Data acquisition and analyses*: M.L.C., N.A., J.H., J.R, R.G.P. *Manuscript revision, supervision, and funding acquisition*: N.S.B., N.A., P.W.G., E.C.H.

## CONFLICT OF INTEREST STATEMENT

PWG and ECH own shares in a company, TroBio Therapeutics, that is developing drugs that target the functions of tropomyosins. The other authors declare no conflicts.

## Supporting information


**DATA S1.** Supporting Information.

## Data Availability

Cells and reagents are available upon request from the corresponding author. Data are present in the manuscript and SI.
