# Supplementary material for: Single molecule visualization of tropomyosin isoform organization in the mammalian actin cytoskeleton
Source: Cytoskeleton (Hoboken). 2024 Jun 14;82(1-2):45–54. doi: 10.1002/cm.21883 (PMC11748362; doi:10.1002/cm.21883)
Supplement: Supplementary file 1 — DATA S1. Supporting Information. [file CM-82-45-s001.pdf]

Supplementary Material

Single molecule visualisation of tropomyosin isoform organization in the mammalian actin cytoskeleton

Maria L. Cagigas, Nicholas Ariotti, Jeff Hook, James Rae, Robert G. Parton, Nicole S. Bryce, Peter W. Gunning and Edna C. Hardeman

APEX2-to-APEX2 distances for Tpm4.2-APEX2 filaments (nm)

Filament

1	2	3	4	5	6	7	8	9	10	11	12	13
33.334	28.07	17.544	36.843	35.088	24.561	28.071	21.053	17.544	35.088	21.052	14.035	17.544
28.07	36.842	21.052	29.824	40.351	19.298	29.824	22.807	15.789	42.105	21.053	10.526	29.825
28.07	36.842	26.316	29.825	26.314	17.544	15.79	21.053	19.298		21.053	22.807	19.298
28.069	22.807	21.053	31.579		29.825	22.807	26.314	24.562		22.807	14.035	35.088
21.054	15.79	15.788			24.561	19.298	21.054			24.561	12.281	14.035
14.035	12.281	14.036			24.562	21.053	31.579			31.579	10.526	
22.807	17.544	29.825			22.807	35.087	14.035			15.79	10.526	
22.807	17.543	31.579			17.544						19.299	

APEX2-to-APEX2 distances for Tpm3.1-APEX2 filaments (nm)

Filament

1	2	3	4	5	6	7	8	9	10	11	12	13
22.994	18.803	21.606	18.803	17.317	24.431	18.824	20.598	20.965	20.201	17.272	19.211	16.561
18.718	10.649	26.143	11.85	14.117	17.553	21.311	14.437	17.361	12.562	13.248	17.788	23.949
15.097	10.867	18.289	14.543	12.629	15.342	9.884	15.763	30.792	22.111	17.27	23.718	21.147
17.378	7.377	13.538	17.669	16.215	20.564	14.346	14.115	10.156	21.019	9.94	27.513	20.637
14.408	13.162	12.211	9.202	28.036	14.171	19.008	15.573	19.654	17.47	12.775	27.276	23.949
16.734	17.747	13.861	7.963	28.096	14.907	17.023	16.169	31.775	20.201	23.421	22.769	14.522
13.604		10.371	10.132	18.486	18.041	18.565	22.409	23.913	17.47	17.041	20.635	20.637
17.528		14.264	16.921		13.699	22.048	25.871	8.189	10.919	17.27	23.718	30.828
			15.377		17.732	11.85	20.173	24.241	15.833	13.958		22.93
			19.569		15.964	12.983	11.551	22.602		14.204		23.44
			17.884		14.107	14.981	14.862	12.775		17.98		17.198
			17.643		14.505	6.179		5.572		11.84		
						11.487		10.482				
						14.85		15.724				
								10.484				
								15.397				
								21.292				
								6.879				
								12.777				

14	15	16	17	18	19
29.333	44.667	26.666	30.667	32.000	14.667
12.000	21.333	32.000	17.333	37.333	18.667
32.667					28.000
26.001					
34.000					
